# SIRT3 elicited an anti‐Warburg effect through HIF1α/PDK1/PDHA1 to inhibit cholangiocarcinoma tumorigenesis

**DOI:** 10.1002/cam4.2089

**Published:** 2019-04-16

**Authors:** Lei Xu, Yang Li, Lixing Zhou, Robert Gregory Dorfman, Li Liu, Rui Cai, Chenfei Jiang, Dehua Tang, Yuming Wang, Xiaoping Zou, Lei Wang, Mingming Zhang

**Affiliations:** ^1^ Department of Gastroenterology Drum Tower Clinical Medical College of Nanjing Medical University Nanjing China; ^2^ Department of Gastroenterology First Affiliated Hospital of Anhui Medical University Hefei China; ^3^ The Center of Gerontology and Geriatrics West China Hospital, Sichuan University Chengdu China; ^4^ Feinberg School of Medicine Northwestern University Chicago IL; ^5^ Department of Gastroenterology, Nanjing Drum Tower Hospital, Affiliated Hospital of Nanjing University Medical School Nanjing University Nanjing China

**Keywords:** Cholangiocarcinoma, Metabolic reprogramming, SIRT3, Warburg effect

## Abstract

Cholangiocarcinoma (CCA) is an extremely invasive malignancy with late diagnosis and unfavorable prognosis. Surgery and chemotherapy are still not effective in improving outcomes in CCA patients. It is crucial to explore a novel therapeutic target for treating CCA. An NAD‐dependent deacetylase also known as Sirtuin‐3 (SIRT3) has been shown to regulate cellular metabolism in various cancers dynamically. However, the biological function of SIRT3 in CCA remains unclear. In this study, bioinformatics analyses were performed to identify the differentially expressed genes and pathways enriched. CCA samples were collected for immunohistochemical analysis. Three human CCA cell lines (HuCCT1, RBE, and HCCC9810) were used to explore the molecular mechanism of SIRT3 regulation of metabolic reprogramming and malignant behavior in CCA. A CCA xenograft model was then established for further validation in vivo. The data showed that SIRT3 expression was decreased and glycolysis was enhanced in CCA. Similar metabolic reprogramming was also observed in SIRT3 knockout mice. Furthermore, we demonstrated that SIRT3 could play an anti‐Warburg effect by inhibiting the hypoxia‐inducible factor‐1α (HIF1α)/pyruvate dehydrogenase kinase 1 (PDK1)/pyruvate dehydrogenase (PDHA1) pathway in CCA cells. CCA cell proliferation and apoptosis were regulated by SIRT3‐mediated metabolic reprogramming. These findings were further confirmed in CCA clinical samples and the xenograft model. Collectively, this study suggests that in the inhibition of CCA progression, SIRT3 acts through an anti‐Warburg effect on the downstream pathway HIF1α/PDK1/PDHA1.

## 1 INTRODUCTION

Cholangiocarcinoma (CCA) is the second most common primary liver malignant disease with poor prognosis.^1^ Although CCA is a rare tumor, the incidence and mortality of CCA drastically increase annually.^2^, ^3^, ^4^ CCA is a highly malignant tumor and is often diagnosed at a late stage due to the limited diagnostic methods. Surgery provides the only potentially curative treatment for CCA, but most patients are not suitable for surgical treatment at the time of diagnosis owing to rapid progression or tumor metastasis.^5^ Moreover, the efficacy of radiotherapy or chemotherapy in improving the prognosis of CCA patients remains unsatisfactory.^6^, ^7^ Accordingly, it is necessary to explore novel therapeutic targets or strategies for CCA.

During the development and progression of the tumor, several studies suggest that cancer cells can reprogram their catabolism and anabolism to obtain sufficient energy and biosynthesis to support cell survival and growth.^8^, ^9^ Cancer cells manage to uptake and utilize glucose extensively by aerobic glycolysis, and this phenomenon is named the Warburg effect.^10^ This metabolic change provides most of the building blocks (lactate and glycolytic intermediates) required for tumor progression, despite the presence of oxygen.^11^ Therefore, it can be a potential therapeutic strategy by reversing the metabolic pattern or an anti‐Warburg effect.^12^, ^13^ Mounting evidence has shown that Sirtuin‐3 (SIRT3), an NAD‐dependent deacetylase, has a dynamic role in regulating cellular metabolism.^14^, ^15^, ^16^ Especially in breast cancer, cervical cancer, and glioblastoma, SIRT3 is considered to be a tumor suppressor.^15^, ^16^, ^17^ However, there are no data available from previous studies regarding the possible biological role of SIRT3 and its underlying molecular mechanism in CCA. Therefore, further research is necessary.

SIRT3‐mediated metabolic reprogramming directly inhibits the Warburg effect via destabilizing hypoxia‐inducible factor‐1α (HIF1α). HIF1α is a transcription factor that regulates the expression of glycolytic‐related genes.^18^ SIRT3 also inhibits tumor progression by targeting the manganese superoxide dismutase (MnSOD), reducing the production of reactive oxygen species (ROS) and genomic instability.^19^ Therefore, the stability of HIF1α is regulated through SIRT3‐mediated mitochondrial metabolism.^18^, ^20^ In previous studies,^21^, ^22^, ^23^ HIF1α increased pyruvate dehydrogenase kinase 1 (PDK1), which limited the amount of pyruvate entering the citric acid cycle, leading to a reduction in mitochondrial oxygen consumption. HIF1α‐mediated PDK1 overexpression decreased the oxidation of glucose and glutamine while promoting the flux of reductive isocitrate dehydrogenase, resulting in the reductive carboxylation of glutamine into citrate for proliferation, indicating metabolic reprogramming.^24^ Pyruvate dehydrogenase (PDH) is a multienzyme complex that regulates carbohydrate and fat metabolism and catalyzes the conversion of pyruvate into acetyl‐CoA by irreversible decarboxylation. Acetyl‐CoA plays an important role in many biological reactions.^25^ PDHA1, as the major component of PDH, can be phosphorylated and inactivated by PDK1.^26^ Phosphorylated PDHA1 inactivates the whole pyruvate dehydrogenase complex (PDC), reduces pyruvate entering into the tricarboxylic acid (TCA) cycle for oxidative phosphorylation, enhances the Warburg effect and promotes tumorigenesis.

In this study, we demonstrated that SIRT3 expression in CCA tissues decreased significantly. SIRT3 could reduce the expression of PDK1 by inhibiting the expression of HIF1α, thereby maintaining the activity of PDHA1 so that the energy metabolism pattern of CCA cells could be reprogrammed. Therefore, SIRT3 acted through the HIF1α/PDK1/PDHA1 downstream pathway in the inhibition of CCA progression via the anti‐Warburg effect.

## 2 MATERIALS AND METHODS

### 2.1 Ethics, consent, and permissions

The experimental procedures involving animal and human samples for this study were approved by the Ethics Committee of Drum Tower Hospital, Nanjing University.

### 2.2 DEGs in CCA samples from TCGA database

The RNA‐Seq data in the CCA samples were obtained from TCGA database using the GDC Data Portal at https://portal.gdc.cancer.gov. The mRNA expression data included nine normal samples and nine matched CCA samples. All sequencing data were publicly available and ethically irrelevant. The DEGs in CCA and normal tissue samples were identified by the edgeR package in Bioconductor. The edgeR package is based on a negative binomial (NB) distribution that corrects the problem of overdispersion in RNA‐Seq data by using the Poisson model and the Bayes procedure. Data with a value of zero were deleted. The genes with fold change >2 were considered to be DEGs with a *P* < 0.01 and a false discovery rate (FDR) < 0.05.

### 2.3 Functional annotation

The Database for Annotation Visualization and Integrated Discovery (DAVID) online tool (https://david.ncifcrf.gov/) was used for functional and pathway enrichment analyses. The potential biological functions and pathways of the high‐ and low‐ expression genes in CCA were detected through the GO and KEGG pathway enrichment assays.

### 2.4 CCA tissue samples and immunohistochemistry

The CCA clinical tissue samples from 26 patients were diagnosed histopathologically at Nanjing Drum Tower Hospital (Nanjing, China). The pathological characterization and clinicopathological staging for the CCA samples were determined based on the International Union Against Cancer (UICC). Protein lysates for Western blot analysis were obtained from six fresh tumor and adjacent tissues. The other 20 CCA tissue samples fixed by formalin were used for immunohistochemistry. The staining intensity was graded as 0 (absent staining), 1 (weak staining), 2 (moderate staining), and 3 (strong staining). The percentage of the staining was divided into five levels: no positive cells were recorded as 0, less than 25% positive cells were recorded as 1, 25%‐50% positive cells were recorded as 2, 50%‐75% positive cells were recorded as 3, and more than 75% positive cells were recorded as 4. The final staining score for each CCA sample was calculated by multiplication, and the value range was between 0 and 12.

### 2.5 Cell culture and reagents

Human CCA cell lines were HuCCT1 (JCRB, Osaka, Japan), HCCC9810, and RBE (Institute of Biochemistry and Cell Biology, Shanghai Institutes for Biological Sciences, Chinese Academy of Sciences, Shanghai, China). HIBEpiC (ScienCell, Carlsbad, CA, USA) was normal human intrahepatic biliary epithelial cell line. The cells were cultured in RPMI‐1640 medium (Invitrogen, Waltham, MA, USA) containing 10%‐20% fetal bovine serum (Biological Industries, Cromwell, CT, USA), penicillin (Invitrogen) (100 U/mL), and streptomycin (Invitrogen) (100 U/mL). BX‐795 (S1274) and CPI‐613 (S1106) were purchased from Selleck Chemicals (Houston, TX, USA). N‐acetyl‐L‐cysteine (NAC, A7250) and Honokiol (H4914) were commercially purchased from Sigma‐Aldrich (St. Louis, MO, USA).

### 2.6 Western blot

The cells were lysed using lysis buffer (Biosharp) to obtain total protein. Next, loading buffer containing 5% 2‐mercaptoethanol was mixed with the lysate. The proteins were denatured at 100°C for 10 minutes before being separated on 8%‐12% sodium dodecyl sulfate‐polyacrylamide gels. After transfer to PVDF membranes (Millipore), the membranes were incubated for 2 hours at room temperature in TBST containing 5% skim milk. Afterward, membranes were incubated with the corresponding primary antibodies overnight at 4°C according to the manufacturer's instructions. After treatment with the appropriate horseradish peroxidase (HRP)‐conjugated secondary antibodies, the membranes were incubated with ECL western blotting reagents (Millipore). The following antibodies were used: SIRT3 (s4072; Sigma), cMYC (ab32072; Abcam), HIF1α (36169; CST, Danvers, MA, USA), PDK1 (5662; CST), p‐PDHA1 (ab92696; Abcam), GAPDH (ab128915; Abcam), Actin (A5441; Sigma), PDHA1 (ab110334; Abcam), GLUT1 (ab115730; Abcam), Anti‐mouse IgG (7076; CST), and Anti‐rabbit IgG (7074; CST).

### 2.7 Apoptosis assay

Cell apoptosis was analyzed using the Annexin V‐fluorescein isothiocyanate (FITC) Apoptosis Detection Kit (556547; BD Biosciences). The collected cells were washed twice with cold PBS and then resuspended in 100 μL Annexin V binding buffer. After incubation with 5 μL FITC‐conjugated Annexin V and 5 μL propidium iodide for 15 minutes in the dark, 200 μL Annexin V binding buffer was added to each tube. Samples were measured by a BD FACSCanto II flow cytometer (BD Biosciences, CA, USA).

### 2.8 Cell viability assay

Cell viability was detected in 96‐well plates with 2 × 10^3^ cells/well using the CCK‐8 colorimetric assay (Dojindo, Minato‐ku, Tokyo, Japan) and cultured for 24, 48, or 72 hours at 37°C with 5% CO2. After treatment for the indicated times, 10 μL CCK‐8 solution was added to each well. Next, the cells were incubated at 37°C and 5% CO_2_ for 1.5 hours. A scanning multiwell spectrophotometer was applied to determine the sample absorbance at 450 nm. The relative cell viability (%) = (absorbance 450 nm of the treated group  absorbance 450 nm of the blank)/(absorbance 450 nm of the control group  absorbance 450 nm of the blank) × 100.

### 2.9 Oxidative phosphorylation analysis

The OCR XF96 Extracellular Flux Analyzer from Seahorse Bioscience, Inc (North Billerica, MA, USA) was used to detect the OCR, which represents oxidative phosphorylation (OXPHOS). CCA cells were seeded in 96‐well XF cell culture microplates (1.0 × 10^4^ cells/well) and then incubated at 37°C and 5% CO_2 _for 24 hours. The medium was replaced with 175 μL/well of XF‐96 running media supplemented with serum‐free RPMI‐1640. The plates were preincubated in an XF Prep Station incubator (Seahorse Bioscience) at 37°C for 20 minutes without CO_2_. The OCR values were obtained by running the XF96 analyzer. Different compounds that modulate mitochondrial respiration were added to each well sequentially: oligomycin (0.5 μmol/L), carbonyl cyanide‐p‐trifluoromethoxyphenylhydrazone (1 μmol/L), rotenone (1 μmol/L), and antimycin A (1 μmol/L). The OCR was determined during specified programmed time periods (three readings each) as the average numbers between the injections of the inhibitors mentioned above. After normalization of the readings by counting the cell numbers in each well, the final data were calculated. The OCR is expressed as pmol/min.

### 2.10 Metabolite analysis

The metabolite extracts were collected. A total of 2 μL metabolite extracts were injected for gas chromatography‐mass spectrometer (GC‐MS) analysis by an Agilent 6980 GC coupled to an Agilent 5973 MS system. The relative abundances of the metabolites were determined by comparing the abundance of each metabolite with internal standards and cell protein standards.

### 2.11 ROS measurement

Cholangiocarcinoma cells were seeded into 6‐well plates (3 × 10^5^ cells/well). After the respective treatment, 5 µmol/L redox‐sensitive probe DCF was incubated for 30 minutes, and the fluorescence intensity was detected immediately by flow cytometry analysis. The experiments were performed in triplicate and repeated three times independently.

### 2.12 Colony formation assay

Cholangiocarcinoma cells were counted and seeded in 6‐well plates at the concentration of 500 cells/well in complete medium containing 10% fetal bovine serum, and treated with 10 μmol/L BX‐795 and/or siSIRT3. After 48 hours, BX‐795 was replaced with complete medium containing 10% fetal bovine serum. After 14 days, the cells were fixed in methanol and stained with 0.1% crystal violet in 25% methanol for 20 minutes. Finally, the colonies with more than 50 cells were counted. The results are presented as the average of the number of colonies counted in each well for each condition.

### 2.13 Cell transfection

siRNAs targeting SIRT3 (5‐CCA GCA UGA AAU ACA UUU ATT‐3), HIF1α (5‐GAAGGAACCTGATGCTTTA‐3), and the negative control siRNA were commercially purchased (RiboBio, Guangzhou, China). The full‐length SIRT3 and HIF1α plasmids were gifts from the Zhao Lab of Fudan University (Shanghai, China). CCA cells were transfected using the Lipofectamine RNAiMax reagent (Invitrogen) according to the manufacturer's instructions.

### 2.14 Isolation and identification of MIBECs

SIRT3 knockout mice were commercially available. The mice were anesthetized, and the portal veins were perfused with type IV collagenase, followed by the removal of the intrahepatic bile duct and digestion with DNase I, pronase E, and type IV collagenase.^27^ The small and uniform cells were cultured in Dulbecco's Modified Eagle's Medium/F12 medium containing 10% heat‐inactivated fetal bovine serum (Invitrogen, Carlsbad, CA, USA), insulin‐transferrin‐selenium (Gibco, Life Tech, USA), 10 ng/mL hepatocyte growth factor (PeproTech, USA), 10 ng/mL epidermal growth factor (PeproTech, USA), 100 U/mL penicillin, and 100 μg/mL streptomycin in a humidified atmosphere of 5% CO2 at 37°C. After attaching the cells to the plastic culture plate pretreated with type I rat tail collagen, the purity and identification of the cholangiocyte preparations were evaluated by immunofluorescence measurement of cytokeratin 19 (CK19).^28^

### 2.15 Xenograft model

A total of 10 female nude mice were purchased from the Department of Laboratory Animal Science, Nanjing Drum Tower Hospital, and subcutaneously inoculated with HuCCT1 cells (3 × 10^6^) resuspended in serum‐free media. Mice were randomly divided into the control group (100 μL NS, n = 5) and the Honokiol group (100 mg/kg diluted in 100 μL NS, n = 5) after the tumors were visible. All mice in both groups received intraperitoneal injections once every 2 days until they were sacrificed. Xenograft tumors were isolated from sacrificed mice after a month of treatment. The tumor volume was measured with a caliper and calculated by the formula, 0.52*length (L)width (W)^2^. Tumor tissues were fixed with 10% neutral formaldehyde or stored at 80°C for subsequent analyses.

### 2.16 Statistics

Statistical analyses were performed with the SPSS 17.0 software. The data were analyzed using a one‐way analysis of variance (ANOVA) followed by post hoc Duncan tests. All the data are presented as the mean ± SEM, *P* < 0.05 was considered to indicate statistical significance.

## 3 RESULTS

### 3.1 Glycolysis is enhanced in CCA

**Figure 1:**
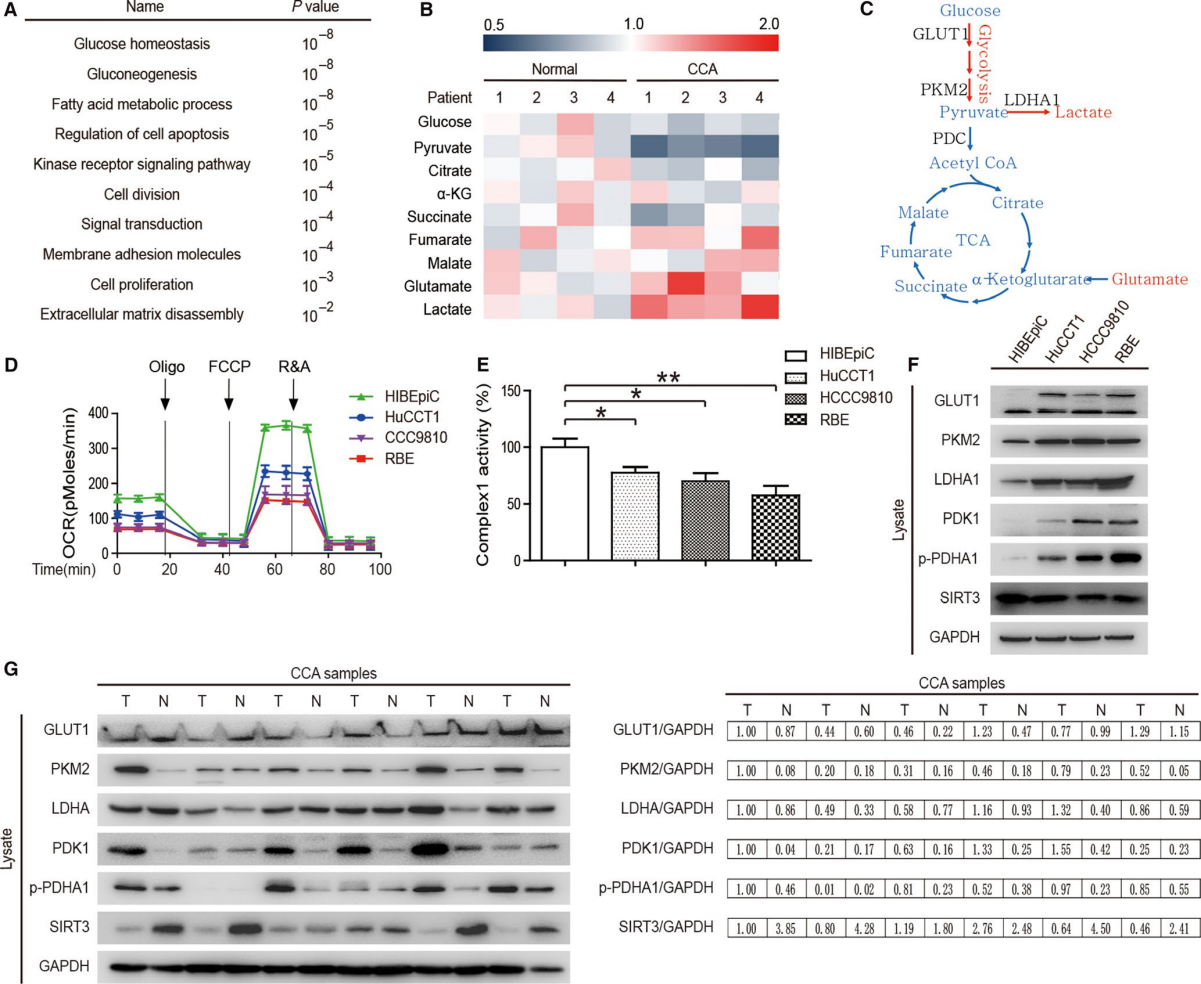
Glycolysis is enhanced in CCA. (A) The differentially expressed genes (DEGs) we screened out in CCA by investigating TCGA database and the most relevant DEGs associated with glucose homeostasis and gluconeogenesis were confirmed through functional enrichment analysis. (B) Tumor tissues and adjacent normal tissues from four CCA patients were collected and lysed, and the concentrations of the metabolites in clinical samples were measured by mass spectrometry (MS). Red indicates high concentration and blue indicates low concentration. (C) A schematic diagram of glucose metabolism suggests an increase in the concentrations of glutamate and lactate and a decrease in the pyruvate concentration. (D) The oxygen consumption rates (OCR) of three different CCA cells and normal intrahepatic biliary epithelial cells (HIBEpiC) were detected at different time points. The OCR under treatments with oligomycin, carbonyl cyanide‐m‐chlorophenylhydrazone (FCCP), and antimycin A/rotenone were detected. (E) Complex1 activity statistics for CCA cells and normal intrahepatic biliary epithelial cells (HIBEpiC). (F) Expression of glucose metabolism‐related enzymes in CCA cells and normal intrahepatic biliary epithelial cells (HIBEpiC). (G) Expression of glucose metabolism‐related enzymes in CCA clinical samples (n = 6). Data represent the mean ± SEM, n ≥ 3. *P < 0.05; **P < 0.01.

Many factors, particularly the tumor microenvironment and hypoxia, can regulate metabolic reprogramming to promote the malignant biological properties of tumors.^29^ Therefore, the differentially expressed genes (DEGs) in CCA were selected via TCGA database, and we confirmed that the most important DEGs were related to glucose homeostasis and gluconeogenesis by functional enrichment analysis (Figure 1A). To further explore glucose metabolism in CCA, four pairs of CCA samples were collected and lysed. The measured concentrations of the metabolites in the CCA samples showed that the pyruvate concentration in tumor tissues decreased while the concentrations of glutamate and lactate increased compared to those of the controls (Figure 1B). In other words, the Warburg effect was significant in CCA with enhancement of glycolysis and inhibition of the TCA cycle (Figure 1C). To analyze the underlying mechanisms, the mitochondrial metabolism of three CCA cell lines (HuCCT1, HCCC9810, and RBE) and one normal intrahepatic biliary epithelial cell line (HIBEpiC) was evaluated. The oxygen consumption rate (OCR) and complex 1 activity of HIBEpiC were higher than those in the other three CCA cell lines (Figure 1D & E). Similarly, the expression of related glycolytic enzymes in HIBEpiC cells was also lower than that of the other three CCA cells. Interestingly, we found increased expression of SIRT3 in HIBEpiC cells (Figure 1F) with higher mitochondrial metabolism (Figure 1D & E). The differences in the expression levels of these molecules were also observed in the clinical samples of CCA (Figure 1G).

### 3.2 Glycolysis is enhanced in SIRT3 knockout mice

**Figure 2:**
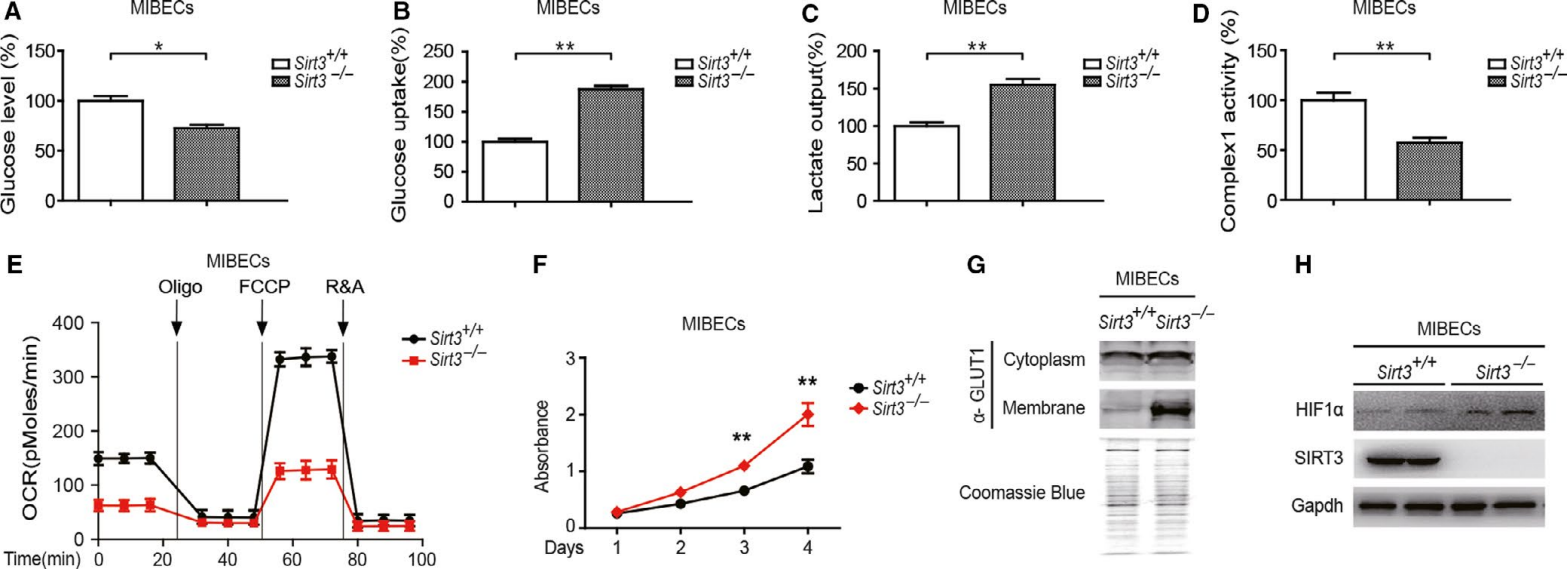
Glycolysis is enhanced in SIRT3 knockout mice. (A) The extracellular glucose levels were detected in MIBECs from control mice or SIRT3 knockout mice. (B) The intracellular glucose uptake was measured in MIBECs from control mice or SIRT3 knockout mice. (C) Lactate output was analyzed in MIBECs from control mice or SIRT3 knockout mice. (D) Complex1 activity was detected in MIBECs from control mice or SIRT3 knockout mice. (E) The OCR of MIBECs from control mice or SIRT3 knockout mice was detected at different time points. (F) MIBECs proliferation from control mice or SIRT3 knockout mice was analyzed via CCK‐8 assay. (G) The cytoplasm and membrane expression of GLUT1 in MIBECs from wild‐type and SIRT3 knockout mice. (H) The expression of HIF1α and SIRT3 in MIBECs from wild‐type and SIRT3 knockout mice. Data represent the mean ± SEM, n ≥ 3. *P < 0.05; **P < 0.01.

The association between SIRT3 and glucose metabolism was further evaluated in SIRT3 knockout mice. Mouse intrahepatic biliary epithelial cells (MIBECs) were separated from wild‐type and SIRT3 knockout mice and cultured in vitro. When analyzing the intracellular and extracellular concentrations of glucose and metabolites in MIBECs, it was found that the extracellular glucose level significantly decreased in SIRT3 knockout mice, (Figure 2A), while the intracellular glucose uptake increased markedly (Figure 2B). Increased glucose uptake led to increased lactate production (Figure 2C) and decreased mitochondrial complex1 activity (Figure 2D). We used the Seahorse bio‐energy analyzer to find a significant reduction of OCR after SIRT3 knockout (Figure 2E). These results suggested that SIRT3 knockout contributed to the upregulated glycolysis and downregulated mitochondrial metabolism. The upregulation of glycolysis means an enhancement of the Warburg effect, therefore, the proliferation of MIBECs from SIRT3 knockout mice was increased (Figure 2F). Glucose uptake is associated with the glucose transporter (GLUT1), which can be regulated by HIF1α.^30^ Next, we found that the membrane expression level of GLUT1 in MIBECs after SIRT3 knockout was significantly increased (Figure 2G). In addition, HIF1α expression was negatively correlated with SIRT3 (Figure 2H).

### 3.3 SIRT3 regulates glucose metabolism in CCA cells through the HIF1α/PDK1/PDHA1 pathway

**Figure 3:**
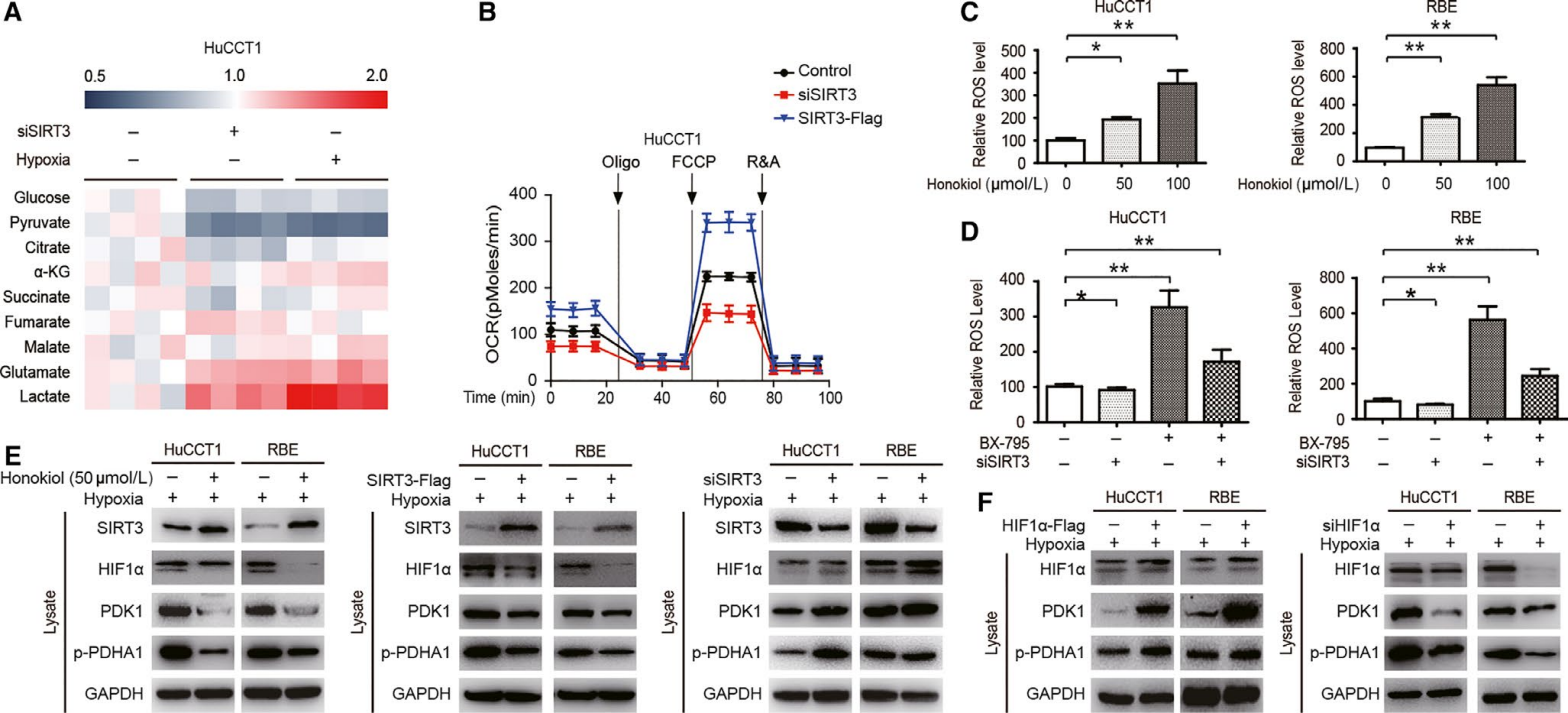
SIRT3 regulates glucose metabolism in CCA cells through the HIF1α/PDK1/PDHA1 pathway. (A) CCA cells in the case of hypoxia or siSIRT3 were collected and lysed, the concentrations of the metabolites in the clinical samples were measured by MS (B) The OCR of CCA cells were detected at different time points following transfection with siSIRT3 and SIRT3‐Flag. (C) Relative ROS levels in HuCCT1 (left) and RBE (right) cells treated with different concentrations of Honokiol for 48 h. (D) Relative ROS levels in HuCCT1 (left) and RBE (right) cells treated with siSIRT3, BX‐795 (50 μmol/L for 48 h), or a combination of siSIRT3 and BX‐795. (E) The key proteins associated with HIF1α/PDK1/PDHA1 in HuCCT1 and RBE cells were assessed following treatment with 50 μmol/L Honokiol for 48 h (left), transfection with the SIRT3 overexpression vector (middle) or siSIRT3 (right). (F) The key proteins associated with HIF1α/PDK1/PDHA1 in HuCCT1 and RBE cells were evaluated following transfection with HIF1α‐Flag (left) or siHIF1α (right). Data represent the mean ± SEM, n ≥ 3. *P < 0.05; **P < 0.01.

The metabolic characteristics in CCA cells consistent with those of MIBECs from SIRT3 knockout mice were observed. The concentrations of glucose and pyruvate decreased, while the concentration of lactate increased in HuCCT1 cells with siSIRT3 or in the presence of hypoxia (Figure 3A). Similarly, when SIRT3 was knocked down, the OCR of HuCCT1 cells decreased correspondingly, and mitochondrial metabolism was downregulated. This effect can be reversed by the overexpression of SIRT3 (Figure 3B). SIRT3 can increase the OCR, indicating increased mitochondrial oxidative phosphorylation, which also induced increases in ROS levels.^31^ The Honokiol derived from the bark of magnolia trees is a natural biphenolic compound, which is also a SIRT3 activator.^32^ We found that the relative ROS levels increased significantly in CCA cells treated with Honokiol (Figure 3C). As HIF1α‐mediated PDK1 overexpression can lead to metabolic reprogramming,^24^ the relative ROS levels in CCA cells treated with PDK1 inhibitors, Ar‐12 and BX‐795, were analyzed and found to also be significantly increased (Figure 3D). This difference can be partially reversed in CCA cells treated with a combination of BX‐795 and siSIRT3 (Figure 3D). To explore potential molecular mechanisms, the expression levels of HIF1α, PDK1, and p‐PDHA1 in CCA cells with SIRT3 upregulation or downregulation were evaluated (Figure 3E). Furthermore, by analyzing the expression of CCA cells with HIF1α overexpression, it was found that the expression of PDK1 and p‐PDHA1 was increased. The downregulation of HIF1α by siRNA can abolish the response (Figure 3F).

### 3.4 PDK1 is a key downstream molecule of SIRT3 regulating CCA cell proliferation and apoptosis

**Figure 4:**
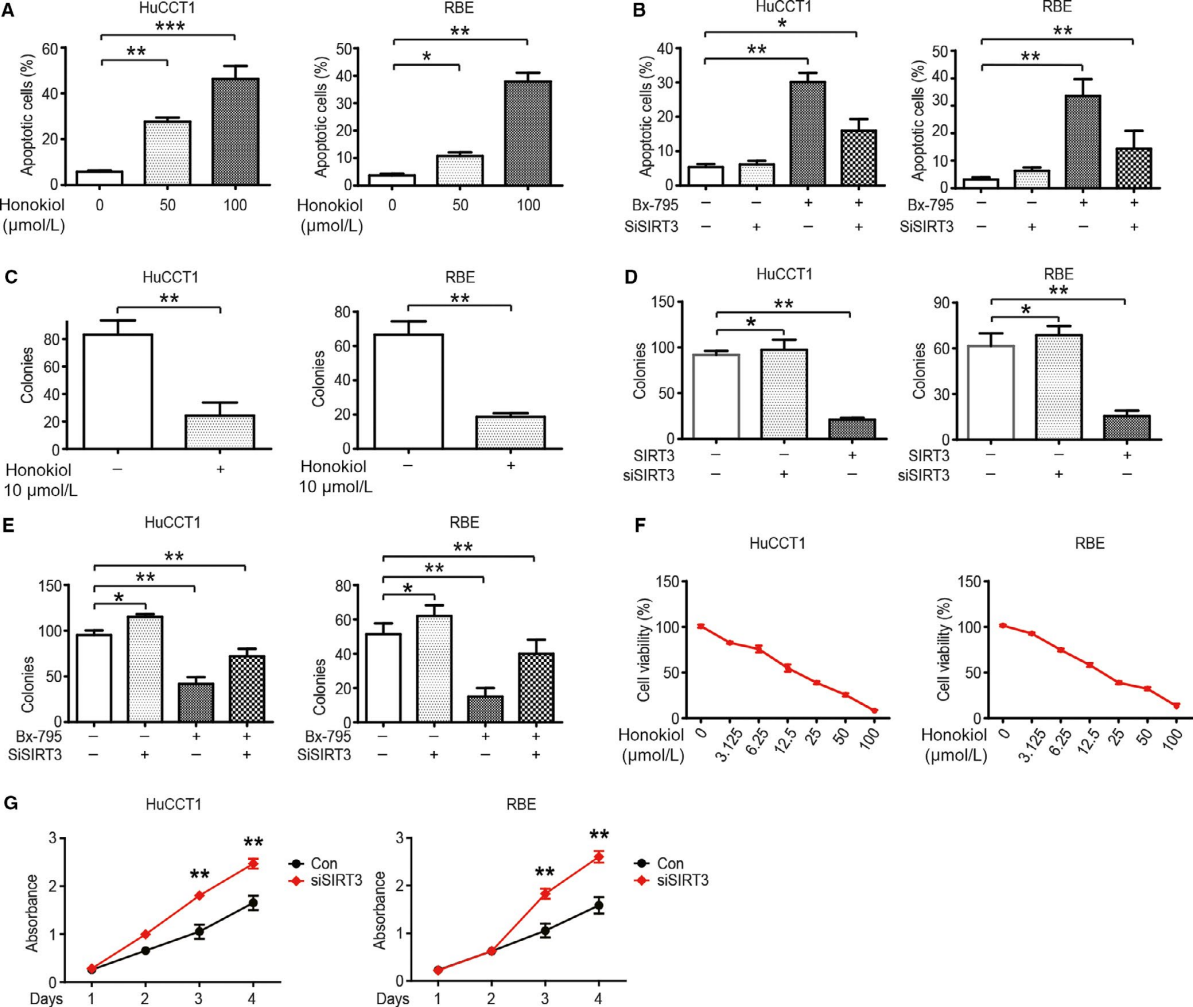
PDK1 is a key downstream molecule of SIRT3 regulating CCA cell proliferation and apoptosis. (A) Apoptotic HuCCT1 (left) and RBE (right) cells were measured via flow cytometry with different concentrations of Honokiol for 48 h. (B) Apoptotic HuCCT1 (left) and RBE (right) cells treated with siSIRT3, BX‐795 (50 μmol/L for 48 h), or a combination of both were analyzed via flow cytometry. (C) HuCCT1 (left) and RBE (right) cells were treated with 10 μmol/L Honokiol for 48 h; cell colonies were quantified after 14 d. (D) HuCCT1 (left) and RBE (right) cells were transfected with siSIRT3 or the SIRT3 overexpression vector; cell colonies were quantified after 14 d. (E) HuCCT1 (left) and RBE (right) cells were treated with siSIRT3, BX‐795 (50 μmol/L for 48 h), or a combination of both; cell colonies were quantified after 14 d. (F) HuCCT1 (left) and RBE (right) cell proliferation was analyzed via the CCK‐8 assay following treatment with different concentrations of Honokiol for 48 h. (G) HuCCT1 (left) and RBE (right) cell proliferation was analyzed via the CCK‐8 assay following transfection with siSIRT3. Data represent the mean ± SEM, n ≥ 3. *P < 0.05; **P < 0.01

SIRT3‐mediated energy metabolism reprogramming can further regulate malignant phenotypes in CCA cells through the HIF1α/PDK1/PDHA1 pathway. Honokiol upregulated SIRT3 activity and promoted apoptosis in CCA cells (Figure 4A). A marginal difference in the apoptosis rate was observed in CCA cells transfected with siSIRT3. However, after inhibition of PDK1 activity by BX‐795, the apoptosis rate of CCA cells was significantly increased, and it was partially reversed by a combination of BX‐795 and siSIRT3 (Figure 4B). The CCA cell proliferation was evaluated through colony formation. It was found that CCA cell proliferation was inhibited after the enhancement of SIRT3 activity by Honokiol (Figure 4C), transfection with the SIRT3 overexpression vector (Figure 4D), and treatment with BX‐795 (Figure 4E). Similar results were obtained from the CCK‐8 assay (Figure 4F & 4G).

### 3.5 SIRT3 inhibits tumor proliferation via the HIF1α/PDK1/PDHA1 pathway in CCA clinical samples and the xenograft model

**Figure 5:**
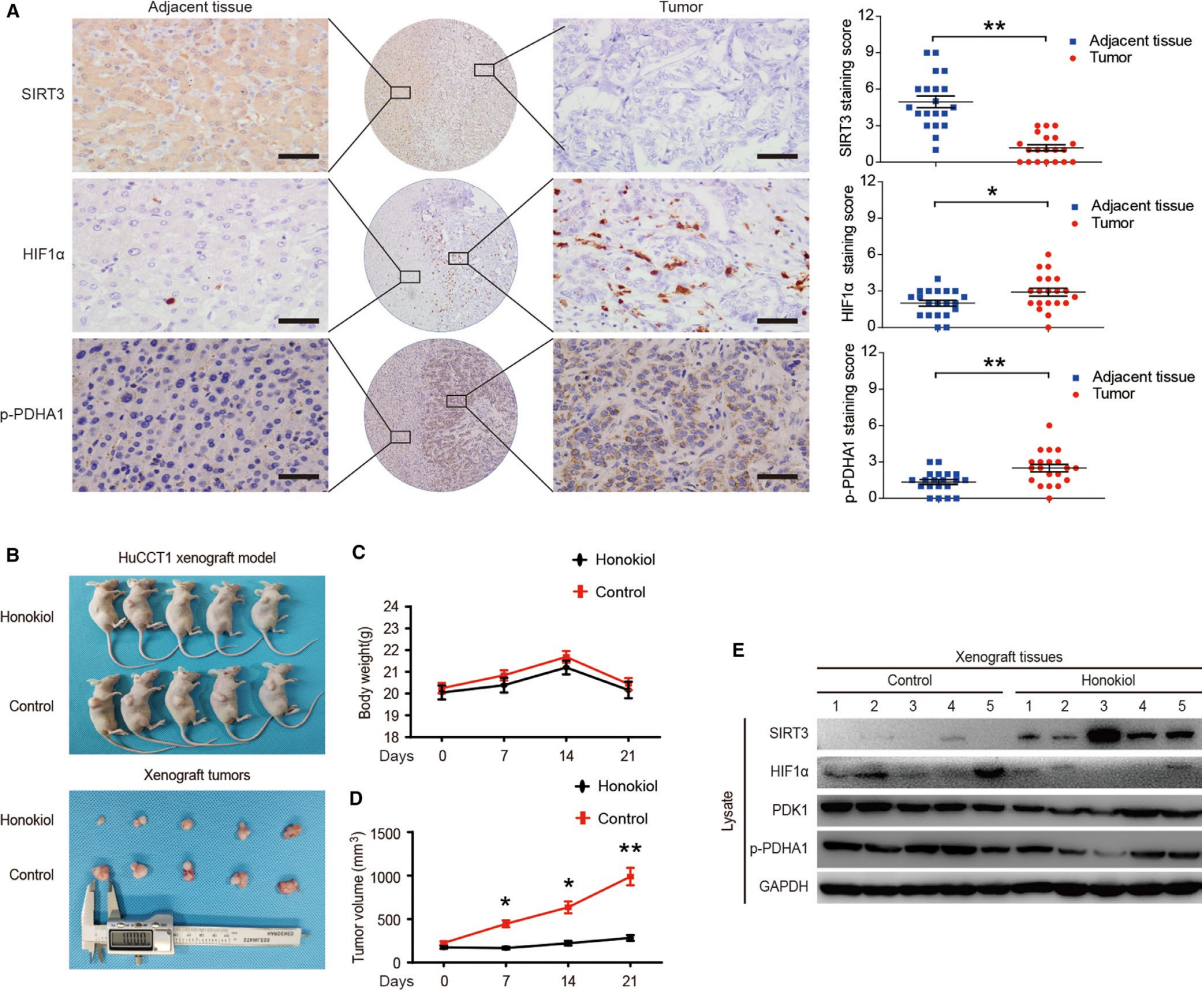
SIRT3 inhibits tumor proliferation via the HIF1α/PDK1/PDHA1 pathway in CCA clinical samples and xenograft model. (A) SIRT3, HIF1α, and p‐PDHA1 protein levels in CCA tumor and adjacent tissues (n = 20) were detected (left) and quantified (right) by immunohistochemistry. The magnification is 400x. Scale bars, 50 μm. (B) Honokiol suppresses CCA cell xenograft tumor growth in nude mice. Tumors were photographed after all animals were sacrificed (n = 10). (C) The body weights of nude mice inoculated subcutaneously with HuCCT1 cells (n = 10). (D) The volume of xenograft tumors (n = 10). (E) Xenograft tumors were isolated and lysed from sacrificed nude mice. The expression levels of SIRT3, HIF1α, PDK1, and p‐PDHA1 were detected by western blotting. Data represent the mean ± SEM, n ≥ 3. *P < 0.05; **P < 0.01; The staining score for each CCA sample was calculated by multiplying the staining intensity by the staining percentage, and the value range was between 0 and 12

Immunohistochemical staining was applied to CCA and adjacent tissues to validate the correlation between SIRT3 and the HIF1α/PDK1/PDHA1 pathway in vivo. Compared with adjacent tissues, the expression of SIRT3 was significantly decreased, while the expression of HIF1α and p‐PDHA1 was significantly increased in CCA tissues (Figure 5A). Next, we established a CCA xenograft model by subcutaneous inoculation of HuCCT1 cells in nude mice (Figure 5B). There was no significant difference in the body weight between the two groups of nude mice (Figure 5C), but the xenograft tumor volume of nude mice in the Honokiol group was significantly smaller than that of the control group (Figure 5D). To further verify the molecular mechanism, xenograft tumors were lysed to detect the expression of related molecules. We demonstrated that Honokiol could activate SIRT3 and downregulate the expression levels of HIF1α, PDK1, and p‐PDHA1 (Figure 5E).

## 4 DISCUSSION

In this study, we found that SIRT3 can reduce aerobic glycolysis and promote oxidative phosphorylation contributing to the Warburg effect in CCA. Thus, inhibiting the development and progression of CCA cells. Furthermore, we found that SIRT3 reverses the Warburg effect by downregulating HIF1α and its downstream PDK1 and p‐PDHA1. Moreover, SIRT3 could be a novel therapeutic target for the treatment of CCA.

SIRT3 is a highly conserved enzyme widely present across species, from bacteria to humans. The removal of the acetyl group from the lysine residues is coupled with the hydrolysis of NAD to generate nicotinamide, lysine, and O‐acetyl‐ADP‐ribose. SIRT3 is responsible for catalyzing many biological processes, including development, genetic control, and metabolism. Previous studies suggested that SIRT3 might play an important role in inhibiting cancer proliferation and progression.^20^, ^33^, ^34^ However, in other tumors, studies have shown that SIRT3 may be a cancer‐promoting gene.^14^, ^35^ These phenomena may be because SIRT3, a deacetylase, has multiple substrates, which allows it to promote tumor progression in one circumstance, and inhibit tumor progression under other circumstances. The acetyl‐proteome is regulated by SIRT3 in core mitochondrial processes that are common to the liver, heart, brain, kidney, and skeletal muscle. However, SIRT3 regulates metabolic pathways differently in tissues producing and utilizing fuel.^36^ Recent studies have shown that by targeting a range of crucial regulators and their relevant pathways in tumors, SIRT3 may perform the role of either tumor suppressor or oncogene by affecting cell growth.^37^ Accordingly, SIRT3 influences the biological behaviors of various tumors through different metabolic regulation patterns.

Post‐translational modifications (PTMs) in metabolism regulation have attracted attention because of their ability to respond to changes in cellular metabolic status and to regulate upstream signaling pathways.^38^, ^39^ SIRT3 is a nicotinamide adenine dinucleotide (NAD^+^)‐dependent mitochondrial protein deacetylase. Acetylation is a crucial modification of proteins in cell biology. Many studies demonstrated that SIRT3 can change the biological function of HIF1α by targeting acetylation regulation.^18^, ^40^ Histone proteins are acetylated and deacetylated on lysine residues at the N‐terminal tail as part of gene regulation. Lysine acetylation regulates protein stability and function. Acetylation of HIF1α (Lys‐709) maintains protein stability and downregulates the polyubiquitination in both normoxia and hypoxia.^41^ We found that SIRT3 overexpression reduced the stability of HIF1α in hypoxic cells, suggesting that SIRT3 directly inhibits the function of HIF1α.

Importantly, previous studies have shown that SIRT3 controls the stability of HIF1α. In addition, SIRT3 induces and regulates the important HIF1α‐targeted genes (such as PDK1) that are related to aerobic glucose consumption.^18^, ^42^ PDK1 can be induced by HIF1α and inactivate the PDH complex by phosphorylation of the PDHA1 subunit at S293. However, the PDH complex plays a key role in the conversion of pyruvate to acetyl‐coenzyme A (acetyl‐CoA) and CO_2_. These result in the inhibition of the tricarboxylic acid (TCA) and pyruvate metabolism cycle‐coupled electron transportation and thus attenuation of ROS production and mitochondrial respiration. Through eliminating pyruvate from mitochondrial consumption, glycolysis and the rate of pyruvate's conversion into lactate may be promoted by PDK1.^43^ PDK1 splicing reporters confirm that transcriptional activation by HIF1α is sufficient to increase exon inclusion of the PDK1 splicing reporter. In contrast, transcriptional activation of a PDK1 minigene by other transcription factors fails to modulate PDK1 RNA splicing without endogenous HIF1α target gene activation.^44^ HIF1α stabilization stimulated glycolysis in bone by upregulating crucial glycolytic enzymes including PDK1.^45^ In glioblastoma multiforme, the inhibition of HIF1α with chrysin reduced the expression of PDK1, PDK3, and GLUT1 and significantly promoted cell death under both normoxic and hypoxic conditions.^46^ Additionally, some studies described that SIRT3 reduces the production of ROS in some other cancers. In the present study, we found that SIRT3 activator increased the ROS levels. The reasons for this contradiction might be that mitochondria are key organelles that regulate the redox homeostasis of both normal and cancer cells. Cancer cells can obtain higher steady‐state levels of ROS counterbalanced by increasing antioxidant capacity than normal cells. Use of metabolic inhibitors can induce tumor cell death by elevating the intracellular ROS levels.^47^ In present study, we demonstrated that SIRT3 inhibited CCA proliferation by inhibiting glycolysis and enhancing mitochondrial metabolism. And the enhanced mitochondrial metabolism can lead to increased ROS levels. Therefore, SIRT3 induced increased ROS levels by promoting mitochondrial metabolism in CCA cells.

Previous studies have shown that the expression of HIF‐1α was correlated with glucose metabolic enzymes in a variety of malignancies. Studies indicated that the expression of HIF‐1α was suppressed and the E‐cadherin protein was increased, while the glycolysis‐related protein PDK1 was downregulated and PDHA1 was upregulated.^48^ Therefore, the expression of HIF1α may influence tumor phenotypes by regulating the Warburg effect. PDK1/PDH regulates the key switch between glycolysis and oxidative phosphorylation in cancer cells, therefore, PDK1 is a crucial target for tumor metabolism in anticancer therapy. Selective inhibitors targeting PDK1 increased the activity of PDH and inhibited the kinase activity of PDK1 directly. In several different cancer cell lines, PDK1 inhibitors can reduce glycolysis in the cytoplasm, downregulate PDH phosphorylation, upregulate the mitochondrial respiration, reverse mitochondrial hyperpolarization, activate several proteins in the apoptotic signaling pathway, and then induce cell apoptosis.^49^ A decrease in PDH expression is important for the aberrant preferential activation of glycolysis in cancer cells under normoxic conditions. Phosphorylation‐dependent inhibition of PDH is a related event in this process. PDH plays a crucial regulatory role by tuning glycolytic metabolism in cancer cells.^50^, ^51^ Enhanced glycolysis inhibited pyruvate dehydrogenase flow, and downregulated TCA cycle labeling, consistent with the Warburg effect.^52^ Furthermore, several studies reported that SIRT3 expression may be correlated with hexokinase‐1 (HK1) and hexokinase‐2 (HK2) expression in the regulation of glucose metabolism in ovarian cancer cells. It remains unclear how SIRT3 regulates cell metabolism by affecting the expression of HK1 and HK2 in CCA, which will be the target of our further study.

In conclusion, SIRT3 expression defects are present in CCA patients. By inhibiting the HIF1α/PDK1/PDHA1 pathway, SIRT3 regulates the expression of pyruvate, lactate, and citrate, increases mitochondrial oxidation, and inhibits extracellular acidification, which corresponds to a decrease in cell proliferation.

## CONFLICT OF INTEREST

The authors declare no conflict of interest.

## AUTHOR CONTRIBUTIONS

Mingming Zhang, Lei Wang, and Xiaoping Zou designed the study. Lei Xu, Yang Li, and Lixing Zhou conducted the cell experiments. Yang Li and Lei Xu collected the tissue samples. Lei Xu, Yuming Wang, and Lixing Zhou performed the protein analysis. Lixing Zhou, Yang Li, Mingming Zhang, and Dehua Tang drafted the manuscript and conducted the immunohistochemistry experiments. Lei Xu, Rui Cai, and Chenfei Jiang performed the metabolite analysis. Robert G. Dorfman and Mingming Zhang wrote the manuscript. Mingming Zhang and Xiaoping Zou supported the study. All authors read and approved the final manuscript.
